# Increased proportion of nitric oxide synthase immunoreactive neurons in rat ileal myenteric ganglia after severe acute pancreatitis

**DOI:** 10.1186/1471-230X-11-127

**Published:** 2011-11-23

**Authors:** Zhong Lin, Ying Liu, Qinghua Zheng, Qinghua Hu

**Affiliations:** 1 Department of Gastroenterology and Hepatology, The Affiliated Hospital of Guilin Medical College, 15 Lequn Street, Guilin 541001, Guangxi Zuang Autonomous Region, People's Republic of China

**Keywords:** severe acute pancreatitis, gastrointestinal dysmotility, enteric nervous system, myenteric ganglion, nitric oxide synthase, neuron

## Abstract

**Background:**

Severe acute pancreatitis (SAP) remains a potentially life-threatening disease. Gastrointestinal motility disturbance such as intestinal ileus is seen in every case. By now, the mechanisms of pancreatitis-induced ileus are largely unknown. The main purpose of the present study was to observe changes of nitric oxide synthase-immunoreactive (NOS-IR) neurons in ileal myenteric ganglia in SAP rats with gastrointestinal dysmotility, trying to explore underlying nervous mechanisms of pancreatitis-induced ileus.

**Methods:**

Twenty Sprague Dawley rats were randomly divided into sham operated group and SAP group. SAP was induced by retrograde cholangiopancreatic duct injection of 5% sodium taurocholate. Abdominal X-ray and intestinal transit were performed to detect the existence of paralytic ileus and intestinal dysmotility. Pathological damage of pancreas was evaluated. Double-immunolabeling was employed for the whole-mount preparations of ileal myenteric ganglia. The morphology of NOS-IR neurons were observed and the percentage of NOS-IR neurons was calculated based on the total Hu-immunoreactive neurons. Total RNA of ileum was extracted according to Trizol reagent protocol. Neuronal NOS (nNOS) mRNA expression was evaluated by RT-PCR.

**Results:**

The small intestinal transit index in the SAP group was significantly lower compared with the sham operated group (29.21 ± 3.68% vs 52.48 ± 6.76%, *P <*0.01). The percentage of NOS-IR neurons in ileal myenteric ganglia in the SAP group was significantly higher than that in the sham operated group (37.5 ± 12.28% vs 26.32 ± 16.15%, *P <*0.01). nNOS mRNA expression in ileum of SAP group was significantly higher than that in the sham operated group (1.02 ± 0.10 vs 0.70 ± 0.06, *P *< 0.01).

**Conclusions:**

The increased quantity of NOS-IR neurons in ileal myenteric ganglia and increased nNOS mRNA expression may suggest nNOS over expression as one of the nervous mechanisms of gastrointestinal dysmotility in SAP rat.

## Background

Severe acute pancreatitis (SAP) remains a potentially life-threatening disease with a mortatity rate about 16.3% [1]. Forty to seventy percent of patients with pancreatic necrosis develop pancreatic infection leading to the development of sepsis, multiple organ failure (MOF) and eventual death [2]. Clinical evidence and animal experimentation of SAP have demonstrated that gastrointestinal motility disturbances such as intestinal ileus play a critical role on pancreatitis-associated sepsis and multiple organ failure [2,3]. Intestinal dysmotility results in gut barrier dysfunction, bacterial overgrowth and bacterial translocation from gut lumen. Enteric bacterial flora is the main source of pancreatic infection in SAP. In clinical practice and experimental SAP, therapies for restoration of intestinal dysmotility using prokinetic agents such as traditional Chinese medicine rhubarb can prevent bacterial translocation and attenuate sepsis in SAP patients [4,5].

By now, the mechanisms of pancreatitis-induced ileus are largely unknown. Experimental studies have revealed that many factors are involved, such as gastrointestinal hormones, neurotransmitters, overproduction of inflammatory mediators, tissue macrophage dysfunction, and endotoxin [6-11]. Such factors are able to alter gastrointestinal motility by direct effect on smooth muscle cells or indirectly by influencing the neuronal circuits involved in peristalsis [6].

Nitric oxide (NO) is an importment nonadrenergic, noncholinergic (NANC) neurotransmitter in the enteric nervous system (ENS). It is generated from L-arginine by neuronal NO synthase (nNOS/NOS-I). NO has a potent inhibitory effect on the smooth muscle in several regions of the gastrointestinal tract [12]. NO may also act as a neuromodulator by facilitating or inhibiting the release of other neurotransmitters within the same nerve ending [13]. Also, there is evidence that NO might act as a second messenger within smooth muscle or interstitial cells of Cajal (ICC) [13,14].

The main purpose of the present study was to observe changes of NOS-immunoreactive neurons in ileal myenteric ganglia and ileal nNOS mRNA expression in rat SAP, trying to explore underlying nervous mechanisms of pancreatitis-induced ileus. In this study, the bile duct perfusion-induced pancreatitis model was employed. The bile duct perfusion-induced pancreatitis model is a well-established, reliable, and highly repeatable pancreatitis model with clinical relevance. Cholangiopancreatic duct infusion with taurocholate is a well-established SAP rat model that induces multiple organ failure involving the lung, kidney, liver, intestine, and brain [15].

## Methods

### Animals and experimental models

Twenty male Sprague-Dawley rats (180-220 g, from Experimental Animal Center, Guilin Medical College) were used in this study. All experiments were approved by the Animal Ethics Committee, Guilin Medical College. The rats were randomly divided into two groups: a sham operated group (n = 10) and a SAP group (n = 10). General anesthesia was induced by an intraperitoneal injection of 1 mg/kg of urethane. The abdomen was then shaved and prepared with 5% povidone-iodine solution. A midline incision was made. Duodenum and pancreas were exposed. SAP was induced by injecting 5% sodium taurocholate solution (1 ml/kg, 0.1 ml/min) retrogradely into the cholangiopancreatic duct using a 4.5 gauge needle. In the sham operated group, pancreas was flipped several times without the duct puncture. After the surgery, duodenum and pancreas was carefully returned to the peritoneal cavity and the incision was closed with continuous sutures. The animals were left to recover for one hour and were then put into metallic cages, one rat per cage, and food and water were not allowed to access for 24 h.

### Abdominal X-ray and small intestinal transit index

24 h after induction of pancreatitis, all rats were subjected to abdominal X-ray examination for identification of presence of gas and fluid in the the gut. For intestinal transit test, 1.5 ml of 1% trypan blue solution was intragastrically gavaged for each rat. 30 min later, rats were sacrificed. The entire small intestine was removed under tension-free state. The small intestinal transit index was calculated as trypan blue containing segment length/total length × 100%, as previously described [11].

### H-E Staining

Pancreas was dissected and fixed overnight at room temperature in a pH-neutral, phosphate-buffered, 10% formaldehyde solution. The tissue was then embedded in paraffin, sectioned at a thickness of 5 μm, stained with hematoxylin and eosin, and coded before examination by a pathologist unaware of the specimen identity. The severity of pancreatic damage was graded by the pathologist using scoring criteria according to references [16]. Briefly, H-E stained sections were graded in a blinded fashion for the extent and severity of edema (0-4), Hemorrhage and fat necrosis (0-4), inflammatory infiltration (0-4) and acinar necrosis (0-4). The scores for each histopathologic parameter were summed up, leading to a minimum score of 0 and a maximal possible score of 16.

### Double-immunolabeling study

From each rat, 1.5 cm segment of the ileum, 2 cm orally to the ileocecal orifice, was collected. The segment was placed in ice-cold Kreb's solution (NaCl, 133.0; KCl, 4.7; CaCl_2_.2 H_2_O, 2.5; MgCl_2_, 1.0; NaHCO_3_, 16.3; NaH_2_PO_4_, 1.4; glocose, 7.8, in mM), opened along the mesenteric border, flushed with ice-cold Krebs and pinned tautly on balsa wood with the mucosal surface down. Specimen was subsequently fixed in Stefanini's solution (a mixture of 2% paraformaldehyde and 0.2% picric acid in phosphate buffer, pH 7.2) for 24 h at 4°C. Whole-mount preparation of the myenteric ganglia were prepared by peeling off the circular smooth muscle layer with fine forceps. The double-immunolabeling study was done by incubating the specimens in 10% normal goat serum and 0.5% Triton X-100 in PBS for 30-60 min at room temperature in order to block non-specific binding of antibodies and to enhance penetration of the antibody. Indirect immunofluorescence method was used for double-immunolabeling. Table 1 summarizes the antibodies used and their dilutions. In this study anti-Hu antibodies were used as general neuronal marker. Tissue was incubated with primary antibodies at 4°C in a humid chamber for 24 h. Each individual primary antiserum was applied separately. After washing in PBS (3 × 10 min), the tissue was incubated for 45 min at room temperature in a humid chamber, with secondary antibodies of goat anti-rabbit-IgG tetramethyl rhodamine isothiocyanate (TRITC)-labeled and goat anti-mouse-IgG fluorescein isothiocynate (FITC)-labeled. After washing in PBS (3 × 10 min), the tissue was mounted on glass slides using phosphate-buffered glycerol (pH 8.2) and examined under an Olympus fluorescent microscrope.

**Table 1:** List of antibodies used in this study

Antibody	Species	Code	Dilution	Source
Primary antibodies				

HuC/HuD	Mouse	A-21271	10 μg/mL	Molecular Probes; Eugene, OR

NOS1	Rabbit	Sc-648	1:100	Santa Cruz Biotechnology; Santa Cruz, CA

Secondary antibodies				

TRITC- conjugated goat anti-rabbit IgG	Goat	ZF-0316	1:200	Zhongshan Goldenbridge Biotechnology; Beijing, CHN

FITC-conjugated goat anti-mouse IgG	Goat	ZF-0312	1:100	Zhongshan Goldenbridge Biotechnology; Beijing, CHN

### Quantitive analysis

The proportion of neurons labeled for NOS were estimated by cell counting on preparations double immunostained for NOS and Hu. The proportion of neurons containing NOS were expressed as a percentage of Hu-IR cells. The following protocol was used: starting from one defined point of the preparation and moving across the slide in a systematic way, at least 200 Hu-immunoreactive neurons from each whole mount were examined.

### RT-PCR studies

From each rat, a 1-cm segment of the ileum, beginning at 4 cm orally to the ileocecal orifice was collected. The segment was immediately immersed and rinsed in ice-cold Krebs solution. The procedures of total RNA extration were according to Trizol reagent protocol of manufacturer's instructions (invitrogen, Life Technologies, Ca, USA). Purified RNA was dissolved in water and the concentration measured by absorbance at 260 nm. RNA quality was determined by running samples on 1.5% agarose-formaldehyde gels stained with ethidium bromide, and RNA concentration and purity were determined by optical density measurements at 260 and 280 nm. RNA samples were uniformly of high quality by these standards.

The procedures of RT-PCR analysis were according to the protocol of manufacturer's instructions (Takara, Dalian, China). First-strand cDNA was synthesized from 4 μg total RNA in a reaction buffer of 20 uL containing 5 × PrimeScriptTMBuffer 4 uL, PrimeScriptTMRT Enzyme Mix | 1 uL, Oligo dT Primer 1 Ul, Random 6 mers 1 uL. The reaction mixture was incubated for 15 minutes at 37°C and then 5 seconds at 85°C. The primer chosen for rat nNOS and β-actin were according to references [17,18]. The sequences of sense and antisense primers for rat nNOS and β-actin are listed in Table 2. We subjected 25 μl of the RT-reaction mixture to an one-tube coamplification of nNOS and β-actin as reference standard and internal control. The PCR amplification protocol of nNOS was as followings: after an initial denaturation at 94°C for 5 min, 35 cycles with 1 min of denaturation at 95°C; 1 min of annealing at 53°C and 1 min of extension at 72°C on a thermal cycler. The PCR amplification protocol of β-actin was as followings: after an initial denaturation at 95°C for 3 min, 40 cycles with 45 s of denaturation at 94°C, 45 s of annealing at 56°C and 45 s of extension at 72°C, the last cycle was followed by an extension step at 72°C for 7 min. Amplified products were electrophoresed on 2% agarose gel in TBE buffer, stained with ethidium bromide, photographed under ultraviolet light, and quantified using bandscan software. For semiquantification, the ratio of the optical density of each PCR product and β-actin was determined.

**Table 2:** Sequences of sense and antisense primers for rat nNOS and β-actins

Enzyme and its sequence	PCR Product, bp	cDNA Position	NCBI Refseq	EMBL Accession No
nNOS				

sense 5'-CCTCTCTGGCCACTAATG-3'	335	331-354	NM-052799	

antisense 5'-GACTACATCGTCAGCCTG-3'		551-571		

β-actin				

Sense 5'-AACCCTAAGGCCAACCGTGAAAAG-3'	241	3541-3558		V01217

antisense 5'-TCATGAGGTAGTCTGTCAGGT-3'		3858-3875		

EMBL: European Molecular Biology Laboratories.

### Data analysis and statistics

SPSS17.0 was employed for statistics. Values are expressed as mean and median and range unless otherwise stated. To compare the differences of Hu-IR neurons and NOS-IR neurons between the sham operated group and SAP group, an arcsine transformation of the percentages was carried out before using Mann-Whintney non-parametric U-test. *P *< 0.05 was considered as statistically significant.

## Results

### Abdominal X-ray and small intestinal transit index

**Figure 1:**
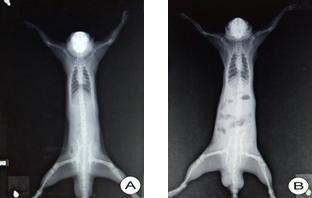
**Abdominal X-ray examination: Gas in intestine is obvious in the SAP rat (B) compared to the sham operated rat (A)**. X-ray film showed small intestinal gas with niveau formation (B).

The intestines in SAP rats were significantly expanded and abdominal X-ray film showed small intestinal gas with niveau formation. Compared with the sham operated group, small intestinal transit index in SAP group was significantly lower (52.48 ± 6.76% vs 29.21 ± 3.68%, *P <*0.01). The results are demonstrated in Figure 1 and Table 3.

**Table 3:** Summary of intestinal transit index and pancreatic pathological score (*t *-test, means ± SEM)

Group	rat	Intestinal Transit index (%)	Pancreatic pathological score
sham operated group	n = 10	52.48 ± 6.76	3.10 ± 0.45

SAP group	n = 10	29.21 ± 3.68*	12.60 ± 1.80*

* *P <*0.01, as compared to sham operated group

### H-E Staining

**Figure 2:**
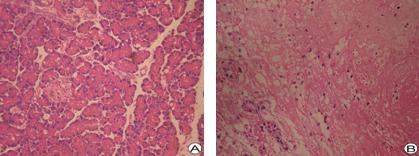
**Pancreatic H-E Staining: Pancreatic tissue displays normal in sham operated group (A)**. In SAP group acinar necrosis and haemorrhage are observed (B). Magnification 20 ×.

All rats in the SAP group displayed pancreatic lesions characteristic of interstitial edema, wide spread acinar cell necrosis, neutrophil infiltration and haemorrhage. In contrast, the pancreas was normal in the sham operated group. The pancreatic pathological score of the SAP group was significantly higher compared to the sham operated group. The results are illustrated in Figure 2 and Table 3.

### The proportion of NOS-IR neurons

Anti-Hu immunoreactivity was present in both nuclei and somal cytoplasm of the myenteric neurons. Neuronal processes were not stained by anti-Hu and the interganglionic connectives were not visible in the whole-mount preparations. This absence of staining of nerve fibres facilitated the identification of individual perikarya and counting of the neurons, making anti-Hu an ideal marker for quantitative analysis on the total numbers of neurons [19,20]. The NOS-IR nerve cell bodies were calculated as percentage of Hu-IR cells. NOS-IR staining was detected in the cytoplasm and in the axons of the neurons. Occasionally, some short lamellar processes arising from the irregular somata profile could also be seen.

**Figure 3:**
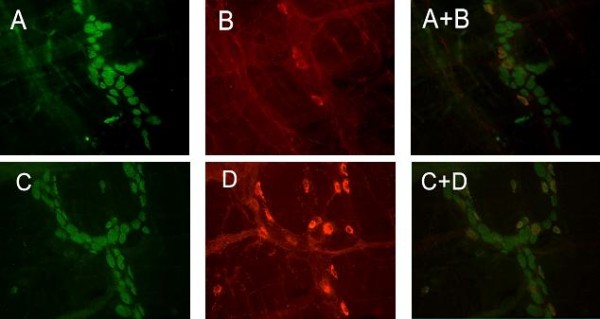
**Double-immunofluorescent staining of whole-mount preparations of myenteric ganglia: the sham operated group (A and B), the SAP group(C and D); Hu-IR neurons (A and C), NOS-IR neurons (B and D); double labelling (A+B, C+D) for Hu (green) and NOS (red)**. Magnification 200 ×.

**Figure 4:**
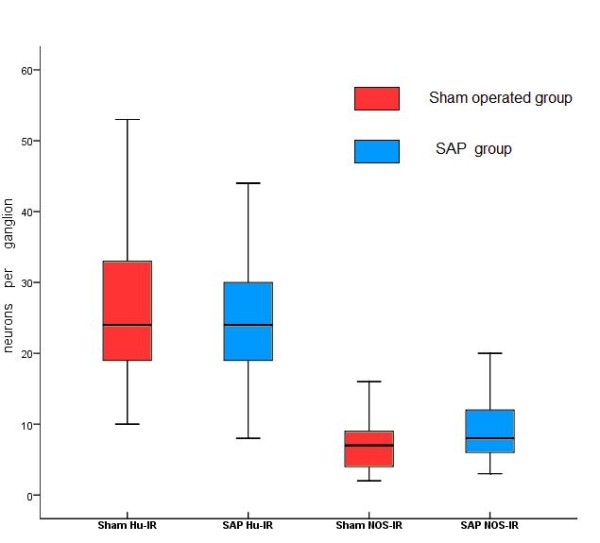
**Compared with sham operated group, the NOS-IR neurons in SAP group increased significantly (*P *= 0.00 *<*0.01), while the Hu-IR neurons did not significantly change in number between two groups (*P *= 0.565). Plots display median (horizontal bars), 25^th ^and 75 ^th ^percentiles (lower and upper limits of boxes), and lowest and highest valves excluding outers (error bars)**.

In sham operated group (n = 10), a total of 105 myenteric ganglia were evaluated. 762 NOS-IR neurons among the 2733 Hu-IR neurons were counted. In SAP group (n = 10), a total of 112 myenteric ganglia were evaluated. 1041 NOS-IR neurons among the 2763 Hu-IR neurons were counted. Statistical analysis using Mann-Whitney nonparametric test (carryed out an arcsine transformation of the percentages before using U-test) showed that the percentage of NOS-IR neurons in the SAP group was significantly higher compared to the sham operated group (37.5 ± 12.28% vs 26.32 ± 16.15%, *P <*0.01). While the Hu-IR neurons in the SAP group did not significantly change in number compared to the sham operated group (24 ± 14.5 vs 24 ± 11.5 per ganglion, *P *= 0.565). The results are summarized in Figure 3 and 4.

### nNOS mRNA expression

**Figure 5:**
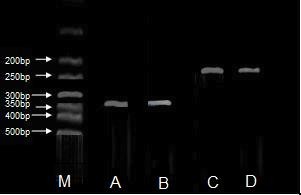
**Epressions of nNOS mRNA in ileum: nNOS of the sham operated group (A), nNOS of the SAP group (B), β-actin of the sham operated group (C), β-actin of the SAP group (D)**.

The expression of nNOS mRNA significantly increased in the SAP group compared to the sham operated group (*P *< 0.05; Figure 5). The ratio of nNOS to β-actin coamplified cDNAs was (1.02 ± 0.10) in the SAP group and (0.70 ± 0.06) in the sham operated group.

## Discussion

In this study, we demonstrated intestinal dysmotility and ileus, by abdominal X-ray and small intestinal transit test, in SAP rats. Double-immunolabeling of NOS and Hu showed that the percentage of NOS-IR neurons in ileal myenteric ganglia in SAP group was significantly higher compared to the sham operated group. nNOS mRNA expression was significantly higher in the SAP rats than in the sham operated rats. These findings strongly suggest that gastrointestinal dysmotility in SAP rats are related to the upregulation of NOS in ileal myenteric ganglia. That may result in an increased production of the inhibitory transmitter NO and possibly also VIP, which finally results in a relaxation of smooth muscle cells and intestinal paralysis.

ENS is a complex of intrinsic intestinal neurons embedded in the gastrointestinal wall. It consists of neurons organized into two main ganglionated plexuses: myenteric ganglia and submucous ganglia. Myenteric ganglia, located between the longitudinal and circular muscle layers in the gastrointestinal wall, mainly regulates gastrointestinal motility. The smooth muscle cells in the circular and longitudinal muscle layers of intestine are innervated by excitatory and inhibitory motor neurons of ENS [21]. Vasoactive intestinal peptide (VIP), NO, pituitary adenylate cyclase-activating polypeptide, noradrenaline and opioids exert inhibitory stimuli. Excitatory stimuli are exerted by tachykinins (for instance substance P, SP), acetylcholine (Ach), and serotonin (5-hydroxytryptamine, 5-HT). The ICC that initiates basic electrical rhythm of gastrointestinal movement also have receptors for the inhibitory transmitters such as NO and VIP and for excitatory tachykinin transmitters such as acetylcholine [22]. Alterations in the balance between inhibitory and excitatory neurotransmitters within the ENS might contribute to enteric motor dysfunction. Genetic primary enteric neuropathic disorders and loss of inhibitory motor neurons to smooth muscle cells bring about dysmotility. Such situations have been demonstrated in the pathogenesis of the following disorders: Hirschsprung's disease, achalasia, irritable bowel syndrome, hypertrophic pyloric stenosis, reflux oesophagitis, gastroparesis, chronic intestinal pseudo-obstruction [21]. Alterations in the balance of SP and VIP are associated with enteric motor dysfunction after small bowel transplantation [23].

NO is a NANC neurotransmitter of ENS generated from L-arginine by neuronal NO synthase (nNOS/NOS-I) that has a potent inhibitory effect on the smooth muscle in various regions of the gastrointestinal tract [12]. NO may also act as a neuromodulator by facilitating or inhibiting the release of other neurotransmitters within the same nerve ending [13]. There is also evidence that NO might act as a second messenger within smooth muscle or interstitial cells of Cajal (ICC) [13,14].

Nervous mechanisms of pancreatitis-induced gastrointestinal dysmotility have been previously demonstrated by other investigators. Mascolo and his colleagues [24] showed that in acetic acid-induced mouse pancreatitis, paralytic ileus and overactivity of cannabinoid CB1 receptors (CB1 receptors) of the enteric cholinergic/SP neurons were observed. Overactivity of CB1 receptors on the enteric cholinergic/SP neurons could lead to reduced release of neurotransmitters of Ach and SP with subsequent delayed motility. Liu and colleagues [25] found that pancreatitis-associated ascitic fluid had effects on the electrogastrointestinal arrhythmia in rats and the mechanisms were related with the activation of nNOS in jejunum myenteric plexus during acute pancreatitis. Our study demonstrated that in the SAP group the percentage of NOS-IR neurons in ileal myenteric ganglia was significantly higher and that nNOS mRNA expression in ileum significantly increased. We hypothesize that the upregulation of NOS results in an increased release of inhibitory transmitters such as NO and alteration in the balance between inhibitory and excitatory neurotransmitters.

Interestedly, Zhou and colleagues [26] reported that the pathogenesis of the small intestinal paralysis in acute necrotizing pancreatitis (ANP) may be related to the deficiencies in ICC and nNOS neurons. The authors observed damages of the interstitial cells of Cajal and myenteric neurons causing ileus in ANP rats induced by intraperitoneal injection of L-ornithine. The authors also observed a reduced number of nNOS-IR neurons in the myenteric ganglia. In Rakonczay's rat models of L-ornithine induced ANP [27], intraperitoneal injection of L-ornithine at the dose of 4 to 6 g/kg killed the animals within hours, and at the dose of 3 g/kg adhesions of abdominal organs were observed. Those factors suggest a toxicity of L-ornithine to cells not only on pancreas but also on neurons of myenteric ganglia. The mechanism underlying L-ornithine-induced pancreatitis is not clear. A decrease in pancreatic polyamine levels is likely to result in an inhibition of DNA and protein synthesis, which will result in the death of acini [27]. It is possible that inhibition of DNA and protein synthesis in the myenteric neurons and direct toxicity of L-ornithine to the myenteric neurons result in a decrease of nNOS-IR neurons. Therefore, different models of acute pancreatitis will result in different changes of myenteric neurons. In our research, we employed the bile duct perfusion-induced pancreatitis model. The mechanism of the bile duct perfusion-induced pancreatitis is attributed to the infusion of exogenous substance, for example taurocholate, and the hydrostatic pressure associated with the infusion [15]. Taurocholate solution is injected into the the cholangiopancreatic duct and has no direct toxicity to the neurons of myenteric ganglia, as the results in our study showed that the Hu-IR neurons in the SAP group did not significantly change in number compared to the sham operated group.

Enteric neuronal plasticity is an essential adaptive response to various injuries or functional changes within the gastrointestinal tract. It is a complex process involving alterations in neuronal excitability, neurotransmitter expression and/or structural rearrangements. These changes can occur transiently in response to an acute stimulus or become permanently encrypted into the enteric neural circuitry following severe injury or a chronic pathological process [28,29]. Evidences of an altered expression of neuropeptides within the enteric neurons in response to neuronal injury, e.g., axotomy, colchicine treatment, isolation and culturing or in response to changes in intestinal activity, are steadily amounting [30,31]. The upregulation of NOS in our study most likely represents a neuronal plasticity reaction to SAP.

In our previous study, changes in the numbers of NOS and VIP expressing neurons were investigated in cultured submucous neurons from rat colon in vitro [32]. The results showed that the proportion of NOS-IR neurons was increased while the proportion of VIP-IR neurons remained unchanged. The possible explanation for this phenomenon is that the VIP-IR neurons may have the ability to synthesize NOS when cultured in vitro. This study suggested an enteric neuron plasticity in given circumstance.

Indeed, ENS is a potential target for pharmacological treatment of gut motor disorders. The targets are numerous, such as the excitatory and inhibitory motor neurones, as well as sensory neurons [21]. Seerden et al [6] reported that tegaserod, a 5-HT_4 _receptor agonist, had a therapeutic benefit in the treatment of pancreatitis-induced ileus. Cerulein, a potent CCK-like decapeptide, activates CCK receptors on enteric neurons and stimulates small bowel motility by releasing excitatory transmitters such as Ach and SP. The pronounced prokinetic effect of clinically used doses of cerulein (0.15-0.3 μg/kg per day) has been confirmed in an experimental setting [33]. Therefore, inhibition of upregulated nNOS mRNA expression using some potential reagents may be a pharmacotherapeutic way to restore the gastrointestinal dysmotility occurring in SAP.

## Conclusions

This study provides with the first evidence of NOS upregulation in ileal myenteric ganglia in rat SAP induced by bile duct perfusion of taurocholate. Gastrointestinal dysmotility was demonstrated in this SAP model by the observation of small intestinal gas with the niveau formation on abdominal X-ray and the lower small intestinal transit index. These findings strongly suggest that gastrointestinal dysmotility in SAP is related to the upregulation of NOS in ileal myenteric ganglia, as NOS is a rate-limiting enzyme of NO production. However, many factors in SAP situation are to be clarified, for example, which neurons are now expressing NOS after SAP and how do the excitatory motor neurons act after NOS upregulation in SAP.

## Competing interests

The authors declare that they have no competing interests.

## Authors' contributions

ZL made the design of the study and paper writing. YL participated in the design of the study and performed RT-PCR test and carried out the immunoassays. QZ carried out the quantitive analysis of myenteric ganglion neurons, performed the statistical analysis and participated in paper writing. QH carried out the animal model making. All authors have read and approved the final manuscript.

## Pre-publication history

The pre-publication history for this paper can be accessed here:

http://www.biomedcentral.com/1471-230X/11/127/prepub
